# New Applicances and Things Medical

**Published:** 1899-11-18

**Authors:** 


### NEW APPLIANCES AND THINGS MEDICAL.

**[We shall be glad to receive, at our Office, 28 & 29, Southampton Street, Strand, London, W.C., from the manufacturers, specimens of all new preparations and appliances which may be brought out from time to time.]**

### TROPON.

(Arthur Reiner and Co., Agents, Dashwood House, (Old Broad Street, London, E.C.)

Tropon is a new albuminous food, consisting of a dry powder which has no taste and no odour. It is not soluble in water, and consequently has to be administered in some form of suspension. Its value lies in its easy administration and its rapid absorption. It is well tolerated by the stomach, even in irritable conditions of the mucous membrane, and hence in gastritis, gastric ulcer, and allied conditions it can be safely relied upon to supply the albuminous requirements of the body. In typhoid fever and in cases of intestinal ulcer, where indigestible *débris* of any kind is dangerous, Tropon will assuredly be found of the greatest service. In various wasting diseases the continued use of this new food has been consistently followed by a marked increase in weight and other signs of improved assimilation. It is a food which should certainly be tried when others have failed; or, better still, it should be the first to be employed.

### ANTISEPTIC, TOILET, AND OTHER SOAPS.

(Edward Cook and Co., Limited, Bow, London, E.)

Biniodide of mercury is perhaps the most popular antiseptic at the present time with the medical profession. Though most destructive in very weak proportions to bacterial life, it cannot be regarded as a very potent poison to human beings; as compared with the uncombined perchloride of mercury it can hardly be regarded as a poison, and yet in its antiseptic properties it is perhaps more active. By skilful technical processes it has been combined in the form of an antiseptic soap, and for use during operations in midwifery practice, and generally by surgeons, it will be found of the greatest use. In dermatological practice, and for skin diseases of microbic origin, it has unrivalled advantages. We have examined several other varieties of soaps prepared by these careful manufacturers. One and all present characters of the highest class, and whether for toilet or general household purposes there are certainly none better on the market. Cook's Savon de Luxe and Riviera soaps, both superfatted and deliciously perfumed, are splendid samples of what can now be manufactured at moderate cost.

